# Seroprevalence and risk factors of bovine fasciolosis in the municipalities of Colombia

**DOI:** 10.14202/vetworld.2023.1293-1300

**Published:** 2023-06-13

**Authors:** Diana María Bulla-Castañeda, Deisy Johana Lancheros-Buitrago, Diego Jose García-Corredor, Julio C-Giraldo-Forero, Martin Orlando Pulido-Medellin

**Affiliations:** 1 Research Group in Veterinary Medicine and Zootechnics, Universidad Pedagógica y Tecnológica de Colombia, Tunja, Colombia; 2 Research Group in Parasitology and Tropical Microbiology, Biology Program, Universidad INCCA de Colombia, Bogotá, Colombia; 3 Research Group in Ecoepidemiology and Collective Health, Faculty of Medicine and Health Sciences, Universidad Militar Nueva Granada Bogotá, Colombia

**Keywords:** bovine, fasciolosis, risk factors, seroprevalence

## Abstract

**Background and Aim:**

Bovine fasciolosis is a reemerging neglected disease with a worldwide distribution caused by the trematode *Fasciola* spp., which parasitize various hosts. Bovine fasciolosis is responsible for large economic losses in the bovine livestock sector. This study aimed to estimate the seroprevalence and risk factors of bovine fasciolosis in the municipalities of Colombia.

**Materials and Methods:**

This was a descriptive cross-sectional study with simple random sampling conducted on 1140 cattle from the municipalities of Chiquinquirá, San Miguel de Sema, and Ubaté for a duration of 3 months. Serum samples were processed using the commercial *Fasciola hepatic*a Antibody Test Kit IDEXX^®^ Fasciolosis Verification (IDEXX, United States), which identified immunoglobulin G antibodies for gf2 antigen purified from *Fasciola* extracts. The f2 antigen is extremely immunogenic and highly specific for *F. hepatica*. An epidemiological survey was performed to record variables related to the sampled animals and herd management practices. Data were processed using the statistical program Epi Info^®^ (Centers for Disease Control and Prevention; Atlanta, Georgia). The prevalence ratio was estimated to evaluate the association between fasciolosis and the hypothesized causal factors and the significance of this association using Pearson’s Chi-square test. Finally, a logistic regression model was developed.

**Results:**

The overall seroprevalence was 72.3%. The seroprevalence was 83.9% (323/385) in Chiquinquirá, 68.17% (257/377) in Ubaté, and 64.55% (244/378) in San Miguel de Sema. The seroprevalence was higher in male animals in Chiquinquirá and in female animals in San Miguel de Sema and Ubaté. Similarly, sex showed a statistically significant association with disease prevalence in Ubaté. The highest prevalence was found in cattle aged >2 years. The Holstein breed showed maximum seroprevalence in Chiquinquirá (p ≤ 0.05) and San Miguel de Sema, whereas crossbreed showed higher seroprevalence in Ubaté. Similarly, in Chiquinquirá, the association between the seroprevalence of fasciolosis and the presence of other species was statistically significant (95% confidence interval [CI]: 0.9601–3.4944; p = 0.0448). In Ubaté, the disease presentation was also associated with pasture rental (95% CI: 0.4047–1.0023; p = 0.003) and attendance to livestock expositions (95% CI: 0.2313–1.0636; p = 0.044). However, in San Miguel de Sema, water from the stream showed a statistically significant association with disease presentation (95% CI: 0.5209–1.0985; p = 0.00649785). Female sex and diarrhea occurrence were considered risk factors for fasciolosis.

**Conclusion:**

A high seroprevalence of antibodies to *Fasciola* spp. was detected in cattle in the study municipalities, indicating a high parasite distribution in these areas. Female sex and diarrhea were established as risk factors associated with fasciolosis in Ubaté and San Miguel de Sema, respectively. Further, research is necessary to establish prevention and control programs against parasitosis.

## Introduction

Fasciolosis is a trematodiasis transmitted by food and water and caused by *Fasciola hepatica* and *Fasciola gigantica* [1, 2]. The disease is considered a reemerging parasitosis of worldwide importance with a major global impact on the health and production of cattle as well as small ruminants [3–5]. *Fasciola* is a parasite capable of infecting a wide variety of domestic and wild mammalian hosts, including humans, and is found primarily in temperate regions [1, 3], where the presence of the snail *Lymnaea* spp. as an intermediate host of *Fasciola* spp. is an essential factor in disease transmission [6].

Infections due to *Fasciola* spp. cause severe economic losses in the cattle sector worldwide, with the global production decline due to fasciolosis reported to exceed US$3 billion per year, which has been associated with detrimental impacts on milk production and quality as well as reduced fertility [7]. According to Utrera-Quintana *et al*. [8], the annual loss caused by the seizure of livers in Mexico was US$7502; the overall production losses were approximately 0.51–1.00 kg of milk per parasitized cow per day, with estimated annual costs of lost milk production per farm ranging from US$ 2218.39 to US$ 6424.51, followed by the costs of anthelmintic treatment for young cattle (US$ 67.68) and adult cows (US$ 209.47) [9]. In Colombia, the existence of endemic zones of *Fasciola* spp. is recognized, including the departments of Boyacá, Antioquia, and Cundinamarca [10–15], and the annual economic losses are approximately $1927 million pesos (US$479,962) [16]. To control this disease, conventional diagnosis has been conducted based on the direct method of detection and egg count in fecal samples after the prepatent period of the parasitosis, where sedimentation techniques improve sensitivity but are not sufficient for early identification of fasciolosis, due to the difficulty of the interpretation of results due to factors such as the long prepatent period and irregular egg shedding [17, 18].

Most infections caused by trematodes are zoonotic and constitute a public health problem. In fact, approximately 600 million animals have been infected with *Fasciola* spp., and 180 million people are at risk of infection worldwide [6]. The World Health Organization estimates that approximately 56 million people worldwide are infected by at least one species of zoonotic trematode [19]. The characteristic clinical signs of bovine fasciolosis include emaciation, fever, hepatomegaly, cholangitis, jaundice, and even death in severe cases [20, 21]. When used individually for the early disease detection, coprological tests are not completely reliable for diagnosing fasciolosis [17, 18]. In this regard, enzyme-linked immunosorbent assays (ELISAs) provide a faster and more sensitive method of serological diagnosis that could detect early acute infection before significant liver damage occurs [17].

Therefore, this study aimed to estimate the seroprevalence and risk factors of fasciolosis in the municipalities of Colombia.

## Materials and Methods

### Ethical approval

This study was approved by Ethics Committee of the Universidad Pedagógica y Tecnológica de Colombia designed by Agreement 001 de 2018, SGI project number 3317 in call for proposal DIN 01-2022.

### Study period and location

**Figure-1:**
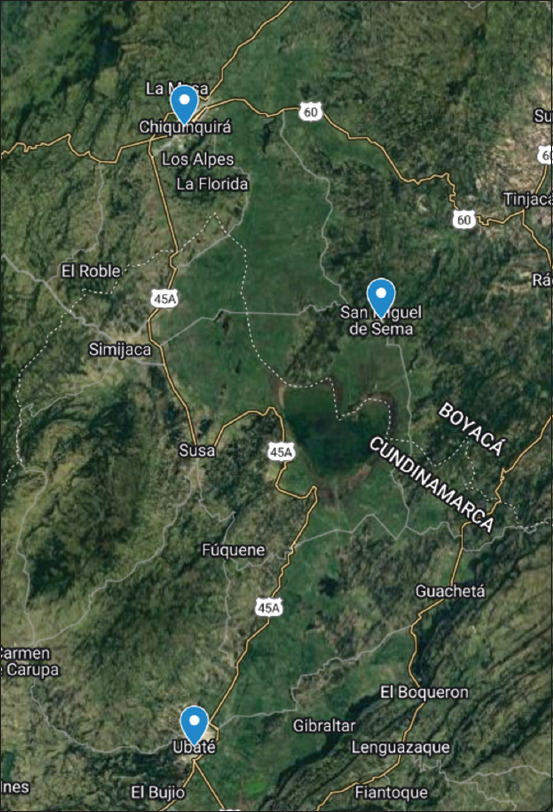
Geographic location of the municipalities of Ubaté, Chiquinquirá and San Miguel de Sema, Colombia [Source: https://www.maps.google.com].

The study was conducted from March to November 2022. The departments of Antioquia, Boyacá, Cundinamarca and Nariño are the areas of the tropics with the highest milk production. In the department of Boyacá, the municipalities of Chiquinquirá, Caldas, San Miguel de Sema and Saboyá reach a volume of 700,000 L per day thanks to the nearly 50,000 cows dedicated to this work, while, in Cundinamarca, the Ubaté Valley, and its entire province, is considered the most dairy regions in the area [22]. Chiquinquirá, San Miguel de Sema, and Ubaté are part of the Ubaté Suárez basin, a region that has been characterized by agricultural activities, mainly dairy farming [23]. Three municipalities were sampled in the study, Chiquinquirá, San Miguel de Sema, and Ubaté, whose geographical locations are Latitude (Lat.): 5.617, Longitude (Long.): −73.8, Latitude (Lat. N.): 5° 37’ 1’’ North and Longitude (Long. W.): 73° 48’ 0’’ West; Lat.: 5.517, Long.: −73.817, Lat. N.: 5° 31’ 1’’, and Lon. W.: 73° 49’ 1’’; and Lat.: 5.30667, Long.: −73.8144, Lat. N.: 5° 18’ 24’’, and Lon. W.: 73° 48’ 52’’, respectively (Figure-1).

The municipalities of Chiquinquirá, Ubaté and San Miguel de Sema have an average height of 2539, 2572, and 2543 m above sea level (masl), respectively, an average temperature of 12°C, 13°C, and 12°C, in addition to having an average rainfall of 1375 mm, 4925 mm, and 2289 mm. A simple descriptive cross-sectional study with simple random sampling was conducted. Cattle were sampled from 65 herds with a zootechnical purpose of milk production and with at least ten cattle; the herds had to have a history of the presence of snails and clinical manifestations associated with Fasciolosis. In addition, the farms were characterized by different water sources for animal consumption (aqueduct, cistern, and stream, among others) and their feed was based on pasture consumption in paddocks, concentrated feed, and supplementation with mineralized salt. On the other hand, the sampled herds had the particularity of having other animal species such as sheep and goats, which grazed in nearby pastures and were sometimes found in the same grazing area.

### Study population and sample collection

According to the National Livestock Census of the Colombian Agricultural Institute, in 2022, the municipalities of Chiquinquirá, San Miguel de Sema, and Ubaté had 33,398, 23,057, and 19,276 heads of cattle, respectively [24]. For our study, a sample size of 385, 378, and 377 female and male dairy cattle aged >6 months was determined for each of the municipalities, respectively, as there have been no studies in these areas, considering a 95% confidence interval, an accepted error of 5%, and an expected prevalence of 50% using the WinEpi statistical program and following the formula mentioned below:


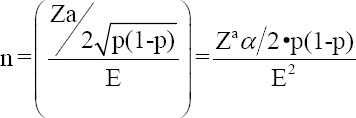


Where: n = sample size; E= accepted error; p = expected value of the proportion; α = tail probability.

A simple descriptive cross-sectional study with simple random sampling was conducted. Blood samples (7 mL) were collected by puncturing the coccygeal vein with 21-gauge needles and a vacutainer-type vacuum tube system with a red cap (BD Vacutainer^®^ Becton, Dickinson, and Company, USA). The samples were labeled, stored, and transported to the Veterinary Parasitology Laboratory of the Universidad Pedagógica y Tecnológica de Colombia (UPTC), where they were centrifuged at 1327.6× *g* for 10 min to obtain serum. Subsequently, the serum samples were stored at −20°C until processing [25].

### Serological detection of anti-*Fasciola* antibodies

The samples were processed by indirect ELISA using the IDEXX^®^ Fasciolosis Verification Test (IDEXX, United States), which identified immunoglobulin G antibodies for gf2 antigen purified from *Fasciola* extracts. The f2 antigen is extremely immunogenic and highly specific for *F. hepatica* (sensitivity 100%, and specificity 57% [26]), according to the manufacturer’s instructions.

### Demographic data

In cattle, risk factors related to the environment have been reported, such as temperature, humidity, water environment such as rivers, and the presence of the intermediate host and factors linked to the definitive host such as age, breed, animal species, and the system of exploitation and feeding management [27]. Considering this information, an epidemiological survey was conducted to record variables related to the animal (sex, age, and breed) and those associated with management practices (presence of cattle from other owners, pasture rental, attendance to livestock expositions, purchase of animals, presence of other species on the farm, deworming, and presence of snail and diarrhea) and the source of drinking water (aqueduct, cistern, stream, and other water sources).

### Statistical analysis

The serological results and the data obtained in the epidemiological survey were consolidated, filtered, and analyzed using the statistical program Epi Info^®^ version 7.2.4.0 (Centers for Disease Control and Prevention; Atlanta, Georgia). The proportions of animals and herds affected by *Fasciola* spp. and exposed to the evaluated factors were compared with the same proportion of a population not exposed to those factors to estimate the prevalence ratio (PR). This PR was used to evaluate the association between fasciolosis and the hypothesized causal factors, as well as the significance of this association using Pearson’s chi-square test [28]. The dependent variable was the serological result of *Fasciola* spp.; the independent variables were all the determinant variables established in the epidemiological survey conducted during sample collection. After establishing these factors, a stratified logistic regression was performed to test the hypothesis and identify the simultaneous interaction between the variables significantly associated with fasciolosis [29].

## Results

The overall seroprevalence was 72.3%. Chiquinquirá showed the highest seroprevalence of 83.9% (323/385), followed by Ubaté (68.17%; 257/377) and San Miguel de Sema (64.55%; 244/378). Regarding sex, in Chiquinquirá, 92.86% (13/14) of male cattle presented antibodies against *Fasciola* spp., whereas female cattle constituted 83.56% (276/310), and in San Miguel de Sema, 65.06% (229/352) of female cattle and 57.69% (15/26) of male cattle were seropositive. In Ubaté, female cattle showed a higher seroprevalence (69.55%; 249/358) than male cattle (42.11%; 8/19). Regarding age groups, cattle aged 2–4 years in Chiquinquirá (87.88%) and San Miguel de Sema (68.14%) showed a higher percentage of antibodies against *Fasciola* spp., whereas cattle aged >4 years were the most prevalent in Ubaté (70.21%) (Table-1).

**Table-1:** Seroprevalence of *Fasciola* spp. by age group in cattle in the municipalities of Chiquinquirá, Ubaté and San Miguel de Sema.

Age groups	n	Positives *Fasciola hepatica*	Prevalence (%)
Chiquinquirá			
<2 years	99	81	81.82
2–4 years	99	87	87.88
>4 years	187	155	82.89
Ubaté			
<2 years	110	72	65.45
2–4 years	32	20	62.50
>4 years	235	165	70.21
San Miguel de Sema			
<2 years	105	67	63.81
2–4 years	113	77	68.14
>4 years	160	100	62.50

Individuals of the Holstein breed showed the maximum seroprevalence in Chiquinquirá (85.64%) and San Miguel de Sema (68.31%), whereas crossbreed showed a higher seroprevalence in Ubaté (Table-2).

**Table-2:** Seroprevalence of *Fasciola* spp. by breed in cattle in the municipalities of Chiquinquirá, Ubaté and San Miguel de Sema.

Breed	n	Positives *Fasciola hepática*	Prevalence (%)
Chiquinquirá			
Ayrshire	105	88	83.81
Cruces	99	80	80.81
Holstein	181	155	85.64
Ubaté			
Ayrshire	76	46	60.53
Cruces	32	25	78.13
Holstein	269	186	69.14
San Miguel de Sema			
Ayrshire	99	63	63.64
Cruces	96	56	58.33
Holstein	183	125	68.31

No statistically significant association was found between seropositivity to *Fasciola* spp. and age groups (p ≥ 0.05) in the three municipalities; however, the Holstein cattle in Chiquinquirá showed a statistically significant association with seropositivity to *Fasciola* spp. (p = 0.023). Moreover, the sex of the individuals in Ubaté showed a statistically significant association with seropositivity to *Fasciola* spp. (p ≤ 0.05), where male sex was considered a protective factor and female sex was considered a possible risk factor for the development of fasciolosis (Table-3).

**Table-3:** Analysis of breed, age groups and sex as possible risk factors associated with Fasciola spp. infections. Results are presented as PR and 95% CI.

Variable	Category	RP	CI (95%)	p-value
Chiquinquirá				
Sex	Female	1.4344	0.0648–2.9120	0.310
	Male	2.3019	0.3434–3.0043	0.321
Breed	Holstein	1.2285	0.7732–1.9520	**0.023**
	Ayrshire	0.9926	0.5955–1.6546	0.544
	Cruces	0.7834	0.4803–1.2777	0.206
Age groups	<2 años	0.8462	0.5140–1.3930	0.306
	2–4 años	1.4423	0.8018–2.5944	0.136
	>4 años	0.8854	0.5611–1.3973	0.350
Ubaté				
Sex	Female	1.9015	1.2566–2.8773	**0.013**
	Male	0.5259	0.3476–0.7958	**0.014**
Breed	Holstein	0.889	0.6494–1.2171	0.271
	Ayrshire	0.8348	0.5197–1.3408	0.296
	Cruces	1.1821	0.8782–1.5912	0.163
Age groups	<2 años	0.889	0.6494–1.2171	0.271
	2–4 años	0.8348	0.5197–1.3408	0.296
	>4 años	1.1821	0.8782–1.5912	0.163
San Miguel de Sema				
Sex	Female	1.2108	0.7560–1.9391	0.288
	Male	0.8259	0.5157–1.3227	0.276
Breed	Holstein	0.9717	0.7193–1.3126	0.471
	Ayrshire	1.1608	0.8496–1.5861	0.202
	Cruces	0.9259	0.6892–1.1889	0.272
Age groups	<2 años	0.9717	0.7193–1.3126	0.471
	2–4 años	1.1608	0.8496–1.5861	0.202
	>4 años	0.9259	0.6892–1.1889	0.272

PR=Prevalence ratio, CI=Confidence interval, Values in bold indicate p ≥ 0.05

In the three municipalities, we detected a statistically significant association between seropositivity to *Fasciola* spp. and the occurrence of diarrhea (p ≤ 0.05). Furthermore, the seroprevalence of fasciolosis was statistically significantly associated with the presence of other species (p = 0.044) in Chiquinquirá, pasture rental (p = 0.003) and attendance to livestock expositions (p = 0.044) in Ubaté, and water from the stream (p = 0.006) in San Miguel de Sema. Diarrhea was considered a possible risk factor for fasciolosis positivity (Table-4).

**Table-4:** Analysis of management practices and water source as possible risk factors associated with *Fasciola* spp. infections. Results are presented as PR and 95% CI.

Variable	Category	RP	CI (95%)	p-value
Chiquinquirá				
Management practices	Presence of other species	1.8186	0.9601–3.4944	**0.044**
	Diarrhea	1.3889	0.8762–2.2015	**0.0107**
Source of drinking water	Stream	0.7568	0.4762–1.2026	0.147
	Aqueduct	0.8542	0.4931–1.4796	0.347
	Cistern	1.0445	0.5458–1.991	0.536
	Other water source	1.3075	0.4423–3.8653	0.439
Ubaté				
Management practices	Pasture rental	0.5384	0.4047–1.0023	**0.003**
	Attendance to livestock expositions	0.4888	0.2313–1.0636	**0.044**
	Diarrhea	0.7544	0.3305–1.0624	**0.000**
Source of drinking water	Stream	1.1483	0.8089–1.6301	0.255
	Aqueduct	0.7598	0.5538–1.0425	0.051
	Cistern	1.2727	0.8703–1.8612	0.123
	Other water source	1.0445	0.5458–1.991	0.536
San Miguel de Sema				
Management practices	Diarrhea	1.3689	1.0061–1.8626	**0.024**
Source of drinking water	Stream	0.6841	0.5209–1.0985	**0.006**
	Aqueduct	1.0767	0.8185–1.4161	0.337
	Cistern	1.0535	0.7612–1.4581	0.426
	Other water source	0.8269	0.4937–1.3851	0.286

PR=Prevalence ratio, CI=Confidence interval, Values in bold indicate p ≥ 0.05

Logistic regression analysis identified female sex as a risk factor for the occurrence of fasciolosis in Ubaté. In contrast, diarrhea was established as a risk factor for seropositivity to fasciolosis in San Miguel de Sema (Table-5).

**Table-5:** Analysis of variables as possible risk factors associated with *Fasciola* spp. infections.

Variable	Odds Ratio	LCI (95%)	SCI (95%)	p-value
Ubaté				
Female	3.1365	1.2276	8.0135	**0.0169**
San Miguel de Sema				
Diarrhea	1.6073	1.0224	2.5268	**0.0398**

SCI=Superior confidence interval, LCI=Lower confidence interval, Values in bold indicate p ≥ 0.05

## Discussion

There is limited published research on the serological diagnosis of fasciolosis in cattle. The previous studies have reported fasciolosis seroprevalence rates of 56% in Uruguay [30], 5.9% in Estonia [31], 9% in Iran [32], and 63.56% in Mexico [9], which are lower than those found in the municipalities evaluated in the present study. However, Alba *et al*. [5] indicated that 90% of the cattle evaluated in Corsica (France) were positive for anti-*F. hepatica* antibodies, showing a wide distribution of the parasite in the area. These variations in seroprevalence may be attributed to increased prevalence and spread of fasciolosis due to climate change, development of drug resistance by the parasite, and intensification of agricultural and livestock practices [4, 33].

Nevertheless, at the national level, the presence of antibodies was established in 5.6% of cattle evaluated in Sabana de Torres (Santander) [34]; 52.4% in animals from the municipalities of El Encino, Duitama, and Belén [35]; 37.8% in 16 municipalities in Colombia [14]; and 13.7% in cattle slaughtered at the Sogamoso (Boyacá) processing plant [13]; these rates were lower than those found in Chiquinquirá, Ubaté, and San Miguel de Sema. The different seroprevalence rates found in our study may be due to the number of bovines sampled, the type of study performed, the ELISA kit used, and the environmental conditions of the geographical location where the study was conducted, because it has been reported that the infection involves a suitable intermediate host in appropriate environments, such as proximity to streams and springs, an optimal temperature between 10°C and 25°C, and sufficient humidity for the disease to occur in ruminants [32, 36].

Regarding the sex of the evaluated animals, studies have reported a greater prevalence of the disease in female animals [14, 30, 37], which is consistent with data found in San Miguel de Sema and Ubaté. However, Ortiz-Pineda *et al*. [13] reported that male animals showed greater seroprevalence, consistent with data found in Chiquinquirá. Furthermore, a statistically significant association was found between the sex of the cattle and the presence of antibodies against fasciolosis in Ubaté, which is in agreement with data reported by Chaouadi *et al*. [37], Pinilla *et al*. [14], and Utrera-Quintana *et al*. [8], where female animals presented a higher risk of parasitic infection, establishing the female sex as a risk factor for the disease. This result could be attributed to stress conditions caused by parturition, lactation, and weaning of offspring, characteristic situations in adult females that cause immunosuppression and increase the rate of parasitic infection [14, 38, 39]. Moreover, the herds in Ubaté were characterized by having extensive production systems with high densities of animals producing milk, unlike those in the farms of Chiquinquirá and San Miguel de Sema, where the production systems were medium and small.

In this study, bovines aged 2–4 years showed a higher percentage of antibodies against *Fasciola* spp. in Chiquinquirá and San Miguel de Sema, and animals aged >4 years showed maximum seroprevalence in Ubaté. These findings were consistent with data reported by Sanchís *et al*. [30], Petersson *et al*. [31], Pinilla *et al*. [34], Chaouadi *et al*. [37], Pinilla *et al*. [35], and Pinilla *et al*. [14], who found the highest seroprevalence in cattle aged >1 year, indicating that the animals of this age group have a higher risk of contracting the parasitic infection, which may occur due to a longer exposure time to the infection and because the life cycle of this parasite requires between 2 and 3 months for the development of the larvae [11, 34, 40]. However, we found no statistically significant association between age groups and the presence of fasciolosis (p ≥ 0.05), which is in contrast to the findings of Chaouadi *et al*. [37], Pinilla *et al*. [35], and Utrera-Quintana *et al*. [8], who did establish this relationship. Moreover, Utrera-Quintana *et al*. [8] mentioned that cattle age was the variable most strongly associated with *Fasciola* spp. infection, which emphasizes the importance of continuous monitoring programs for infection by this trematode and the prevention of economic losses in livestock production. In addition, postmortem studies have shown that older animals have higher levels of liver injury. When the degree of liver injury is high, there is a greater probability of observing the presence of the parasite in the animal liver [41].

The Holstein breed showed the most seroprevalence in Chiquinquirá and San Miguel de Sema, which is consistent with Sanchís *et a*l. [30], Pinilla *et al*. [14], and Pinilla *et al*. [35], who reported that cattle of dairy breeds and pure breeds are more likely to show a higher infection rate to the parasite. This could be because these individuals are more frequently found in cold thermal floors (2000–3000 masl), where high prevalence rates of parasitosis have been reported and where environmental conditions are favorable for the development of the snail [42], which is also related to the association found between seropositivity to the disease and the Holstein breed in Chiquinquirá. Random-effects logistic regression analysis confirmed that animals from mixed herds and beef herds had higher odds of being positive to the serological test detection of antibodies against *F. hepatica* than animals from dairy herds (Table-2). A similar pattern was observed at herd-level (Table-4). Petersson *et al*. [31] mention in their research that animals from mixed and beef herds were more likely to be seropositive for *Fasciol*a spp. a than animals from dairy herds. However, it is difficult to compare results from studies applying different methods and category definitions. Differences in farm management, including aspects such as access to pasture and length of grazing season, are most likely responsible for differences in exposure to *F. hepatica* by production type, but these could not be investigated further in this study.

In our study, the variables presence of other species in the herds in Chiquinquirá and pasture rental and attendance to livestock expositions in Ubaté showed a statistically significant association with the seroprevalence of fasciolosis. It has been reported that different climatic, environmental, and management factors are significantly associated with a higher risk of trematode infection [9]. Moreover, fasciolosis is transmitted between mammalian hosts such as buffaloes, cows, sheep, and other wild ruminants (or humans) as definitive hosts and freshwater *Lymnaea* spp. snails as intermediate hosts [43]. Furthermore, Pinilla *et al*. [35] and Pan *et al*. [6] mentioned that infected snails could be an indispensable risk factor for the transmission of fasciolosis; therefore, coming into contact with other parasitized species and consuming forage in pastures where the history of parasitism is unknown could increase the risk of infection.

In addition, it has been reported that the prevalence of *Fasciola* spp. depends on the proportion of pastures that are implemented in livestock and animal husbandry variables, due to the high infection rates by the trematode in degraded forages (excess livestock and overgrazing) and the parasite benefits from their degradation [44]. Nevertheless, the successful spread of fasciolosis observed currently is primarily associated with the parasite, which is transmitted through consumption of forage or water contaminated with metacercariae [5], probably supporting the fact that the variable stream as a source of drinking water for cattle could be statistically significantly associated with the presence of antibodies against *Fasciola* spp. This is because it has been reported that animals in extensive management systems may be forced to graze in areas such as riverbanks or streams, which are heavily infested with snails [45].

The clinical manifestations associated with the presence of *Fasciola* spp. are generally highly variable, ranging from digestive changes such as diarrhea or inappetence to more general symptoms such as low body condition, anemia, and edema [46]. Hence, diarrhea was established as a risk factor for the occurrence of the disease in the cattle evaluated, indicating that cattle with diarrhea have a 1.6073 higher probability of being positive for the parasite. Therefore, a judicious anthelmintic treatment regimen is recommended to control the disease in endemic regions with the awareness of practices by farm owers to avoid grazing animals along water bodies infested with snails [4].

## Conclusion

This study demonstrated a high seroprevalence of antibodies against *Fasciola* spp. in cattle in the evaluated municipalities, indicating a high parasite distribution in the area. Cattle aged >2 years showed the maximum seroprevalence, indicating that adults have a higher risk of infection, possibly due to a longer exposure time. Female sex was found to be a risk factor for parasitosis in Ubaté, which can be attributed to the stressful situations to which cows are subjected in production systems. Diarrhea is one of the digestive changes caused by fasciolosis, establishing it as a risk factor in San Miguel de Sema. However, it is necessary to consider that the symptoms depend on the state of infection and the parasite load. Further, investigation is required to establish prevention and control programs against parasitosis.

## Authors’ Contributions

DMB, DJL, DJG, JC, and MOP: Contributed to the planning and development of the study and reviewed the final version of the manuscript. DMB and DJG: Performed formal analysis and data curation. DMB and DJL: Drafted the manuscript. All authors have read, reviewed, and approved the final manuscript.
